# Notch signaling-modified mesenchymal stem cells improve tissue perfusion by induction of arteriogenesis in a rat hindlimb ischemia model

**DOI:** 10.1038/s41598-021-82284-3

**Published:** 2021-01-28

**Authors:** Shusaku Maeda, Shigeru Miyagawa, Takuji Kawamura, Takashi Shibuya, Kenichi Watanabe, Takaya Nakagawa, Akima Harada, Dai Chida, Yoshiki Sawa

**Affiliations:** 1 Department of Cardiovascular Surgery, Osaka University Graduate School of Medicine, 2-15 Yamadaoka, Suita, Osaka 565-0871 Japan; 2 SanBio, Inc., Tokyo, Japan

**Keywords:** Stem cells, Cardiology

## Abstract

Notch signaling-modified human mesenchymal stem cell, SB623 cell, is a promising cell therapy product for ischemic stroke. With the aim to expand indications for their use for critical limb-threatening ischemia (CLTI), we hypothesized that SB623 cells improved tissue perfusion by inducing angiogenesis or arteriogenesis in a hindlimb ischemia model rat. In Sprague–Dawley rats, hindlimb ischemia was generated by femoral artery removal, then seven days after ischemic induction 1 × 10^5^ SB623 cells or PBS was injected into the ischemic adductor muscle. As compared with the PBS group, tissue perfusion was significantly increased in the SB623 group. While capillary density did not vary between the groups, αSMA- and vWF-positive arterioles with a diameter  > 15 μm were significantly increased in the SB623 group. Whole transcriptome analysis of endothelial cells co-cultured with SB623 cells showed upregulation of the Notch signaling pathway as well as several other pathways potentially leading to arteriogenesis. Furthermore, rat muscle treated with SB623 cells showed a trend for higher ephrin-B2 and significantly higher EphB4 expression, which are known as arteriogenic markers. In the hindlimb ischemia model, SB623 cells improved tissue perfusion by inducing arteriogenesis, suggesting a promising cell source for treatment of CLTI.

## Introduction

Patients with critical limb-threatening ischemia (CLTI) have a very poor prognosis, mainly associated with unhealed ulcers, limb amputation, and impaired walking function^1^. Although revascularization is essential in treatment of CLTI, approximately 20–30% of those patients are not considered qualified for endovascular treatment or bypass surgery^2,3^. As a promising alternative option, cell therapy has been under development for more than 20 years^4^. In clinical trials, cell therapies have shown some impact on outcomes, including pain relief, ulcer healing, and increase in ankle-brachial pressure index, though response of severely damaged limbs with intense ischemia has not been sufficien ^4,5^. To address the current situation, a new cell therapy product is required, though development requires a great deal of effort and cost to establish safety, accessibility, and stable production. Thus, expansion of indications for cell therapy products for treatment of other diseases is an attractive option.

Notch signaling-modified human mesenchymal stem/stromal cells (hMSCs), SB623 cells, is a promising cell therapy product for ischemic stroke^6,7^. In a phase 1/2a study of chronic stroke patients, two-year safety and improved clinical outcomes were shown. Allogeneic transplantation of SB623 cells and a cell production line has been established. In addition, Notch signal is known as a key regulator of arteriogenesis as well as neural cell differentiation^8–13^. A previous report suggested that not only neurotrophic factors, but also secretion of angiogenic and arteriogenic cytokines were higher in SB623 cells as compared to hMSCs from the same donors^14^. Taken together, SB623 cell is a promising new cell therapy product candidate for CLTI. With the aim to expand indications for their use, in this study, we hypothesized that SB623 cell transplantation improved tissue perfusion by inducing angiogenesis or arteriogenesis in hindlimb ischemia model rats.

## Results

### Chronic hindlimb ischemia model

**Figure 1:**
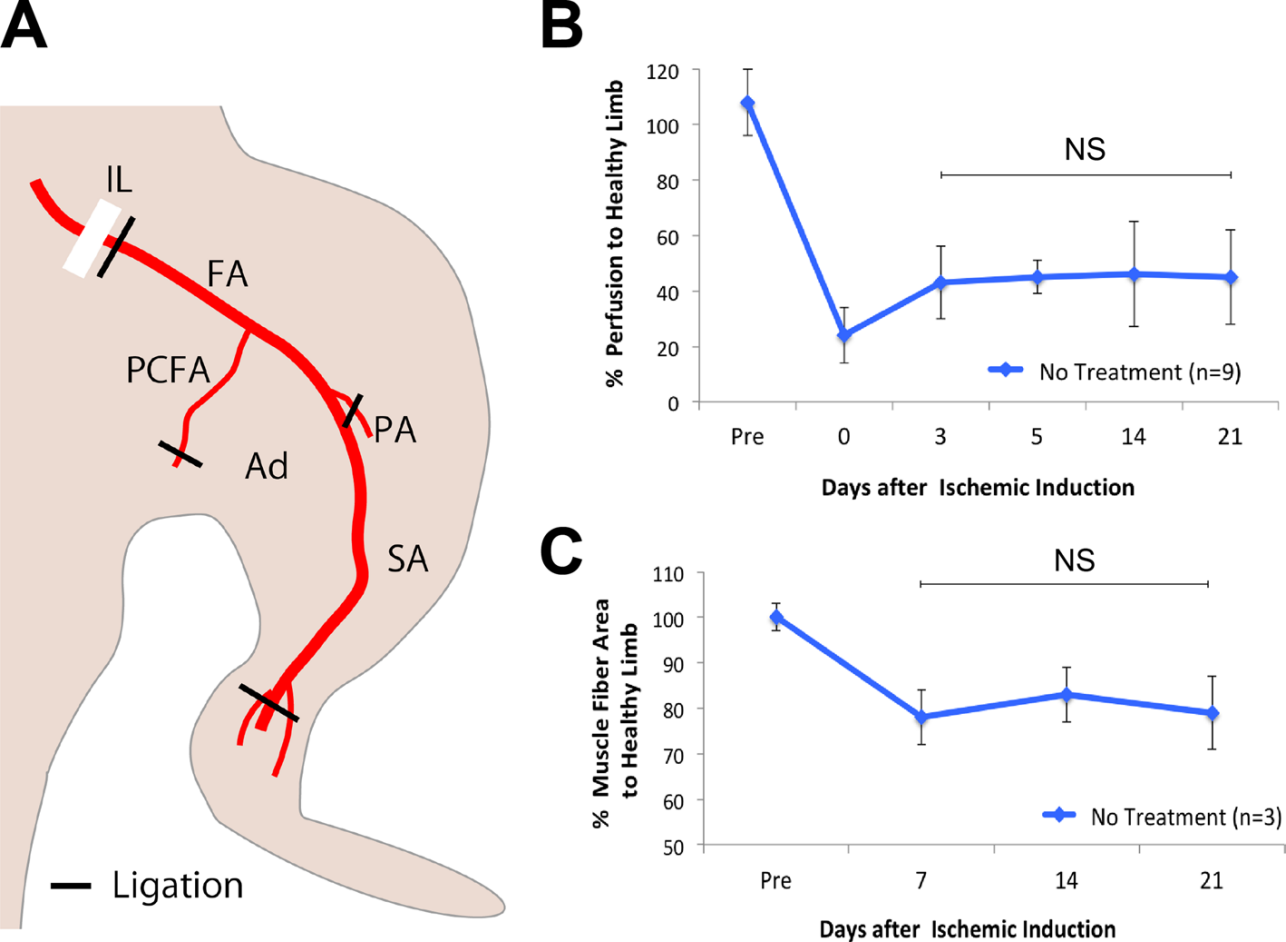
Chronic hindlimb ischemia model. (**A**) Hindlimb ischemia model was created by extended removal of left femoral artery (FA). Proximally, FA immediately below iliac ligand (IL) was ligated. Distally, the most distal portion of saphenous artery (SA) and proximal caudal femoral artery (PCFA) was ligated. Ad, adductor muscle; PA, popliteal artery. (**B**) Natural course of ischemic limb tissue perfusion after ischemic induction. (**C**) Natural course of muscle atrophy after ischemic induction.

The hindlimb ischemia model used in this study was created by extended removal of a hindlimb artery (Fig. 1A). Tissue perfusion was assessed with a laser Doppler perfusion image (LDPI) analyzer (Moor Instruments, Axminster, UK) and the LDPI index was calculated as the ratio of perfusion of ischemic hindlimb to that of healthy hindlimb. At 5 days following ischemia induction, ischemic hindlimb perfusion was decreased to approximately 40%, and then remained unchanged at 2 and 3 weeks (Fig. 1B). Furthermore, muscle atrophy of ischemic limb was assessed histologically. Muscle fiber area calculated as the ratio of muscle area to number of muscle fibers was decreased at 1 week and then was unaltered at 3 weeks (Fig. 1C). These results suggested that the amount of natural recovery of tissue perfusion and muscle atrophy after 1 week was scant in the present model.

### SB623 cell transplantation increased hindlimb tissue perfusion by arteriogenesis

**Figure 2:**
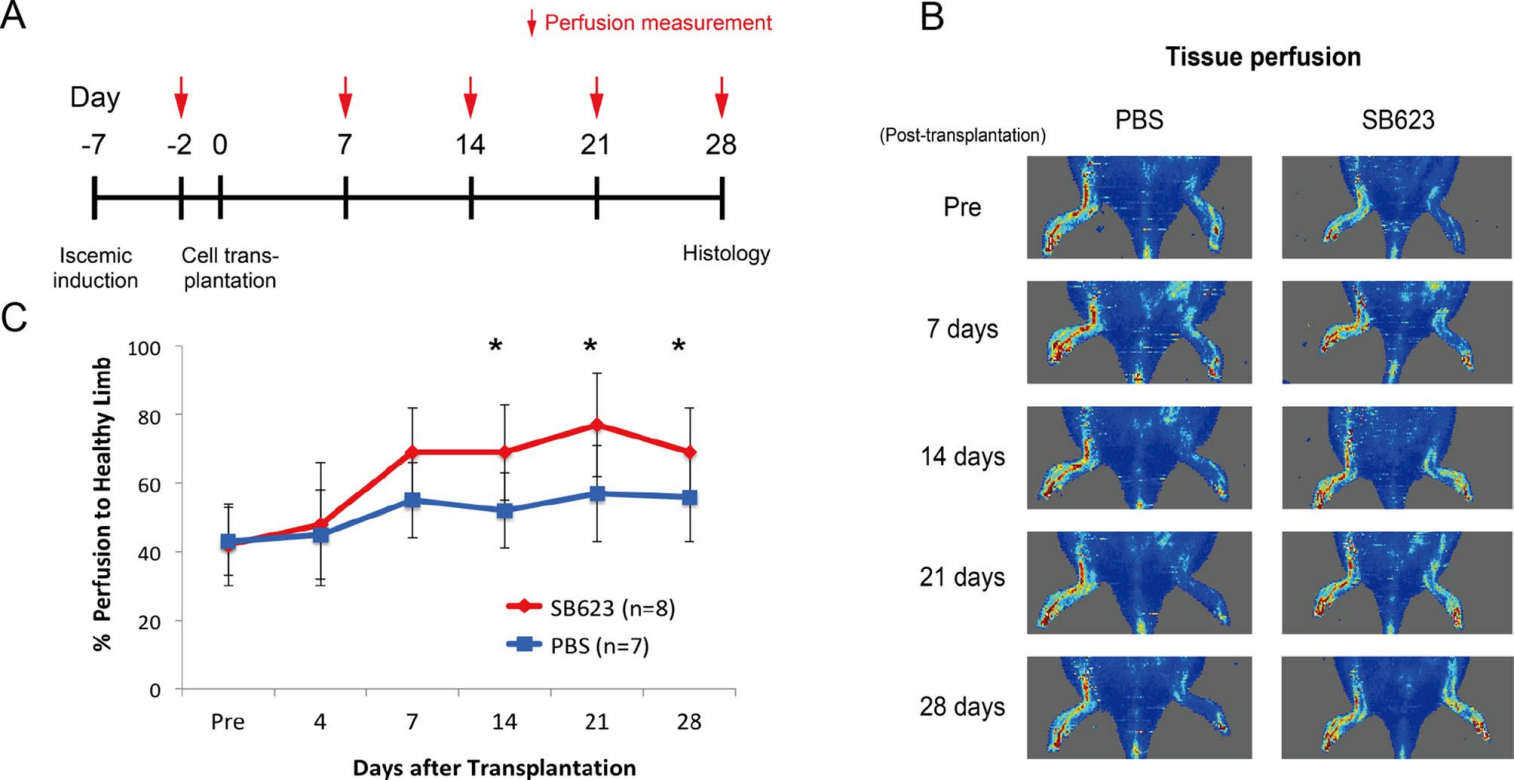
Tissue perfusion measured by laser Doppler. (**A**) Study timeline. (**B**) Representative images of tissue perfusion for control (PBS) and treatment (SB623) groups. (**C**) Ischemic hindlimb perfusion measurements after cell transplantation in each group. *p < 0.05.

We assessed the effects of SB623 cell transplantation on tissue perfusion in our rat model. At 7 days after ischemia induction, 1 × 10^5^ SB623 cells (SB group) or PBS (PBS group) were injected into adductor muscle, then tissue perfusion was assessed over the following 28 days (Fig. 2A**)**. Representative serial perfusion images from each group are shown in Fig. 2B. As compared to the PBS group, hindlimb tissue perfusion in the SB group was significantly improved, with the difference in LDPI index reaching statistical significance (*p* = 0.003) at 2 weeks after cell transplantation (Fig. 2C). In the PBS group, one rat died and one developed gangrene in the ischemic hindlimb during the observation period, whereas there were no death or gangrene in the SB group.

**Figure 3:**
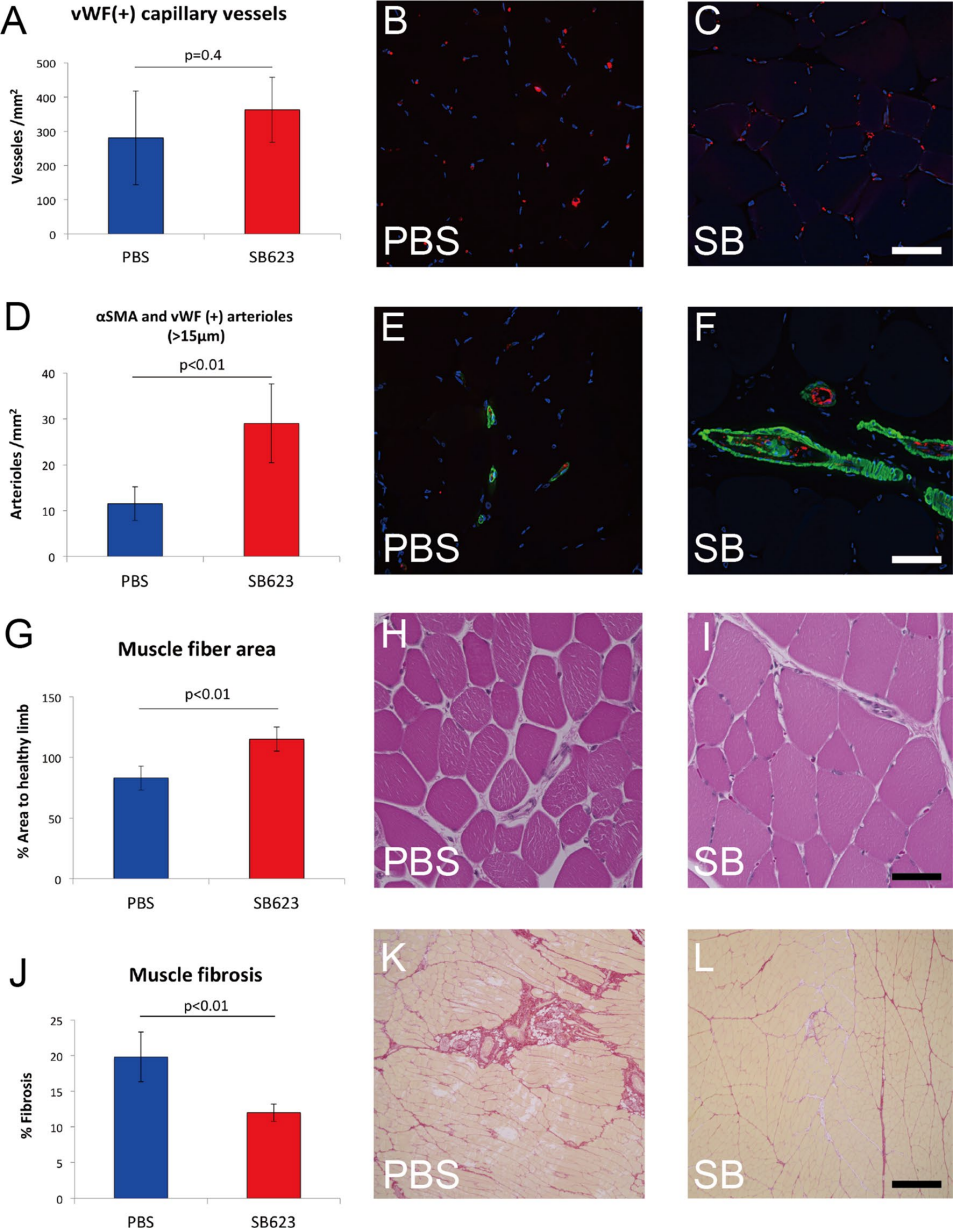
Histological assessment of ischemic hindlimb muscle. (**A**) Density of capillary vessels. (**B**, **C**) Immunostaining for capillary vessels with anti-vWF (red) and nuclei (blue). (**D**) Density of arterioles with a diameter > 15 μm. (**E**, **F**) Immunostaining for arterioles with anti-vWF (red), anti-αSMA (green), and nuclei (blue). (**G**) Muscular atrophy quantification. (**H**, **I**) Muscle fibers stained with Hematoxylin and eosin. (**J**) Interstitial fibrosis quantification. (**K**, **L**) Interstitial fibrosis stained with Sirius red. Scale bars in (**B**)–(**I**) indicate 50 μm and in (**K**)–(**L**) indicate 500 μm.

Capillary and arteriole numbers were analyzed on tissue cross-sections of adductor muscle at 4 weeks after cell transplantation. In the SB group, the number of capillary vessels was relatively increased, while it was not statistically significant (*p* = 0.36) (Fig. 3A). Representative images of capillary staining are shown in Fig. 3B, C. On the other hand, the number of mature arterioles with a diameter > 15 μm was significantly increased in the SB group (*p* = 0.006) (Fig. 3D), with representative images of arteriole staining shown in Fig. 3E, F.

### SB623 cell transplantation attenuated muscle atrophy and interstitial fibrosis

Muscle ischemic damage was analyzed on tissue cross-sections of adductor muscle. At 4 weeks after cell implantation, the muscle fiber area in the PBS group was decreased as compared to that in the SB group (*p* = 0.002) (Fig. 3G), suggesting that SB623 cell transplantation attenuated muscular atrophy caused by ischemia. Representative images of hematoxylin and eosin staining are shown in Fig. 3H, I. In addition, interstitial fibrosis was significantly decreased in the SB group (*p* = 0.004) (Fig. 3J). Representative images of Sirius red staining are shown in Fig. 3K, L.

### Molecular mechanism of arteriogenesis induced by SB623 cell transplantation

**Figure 4:**
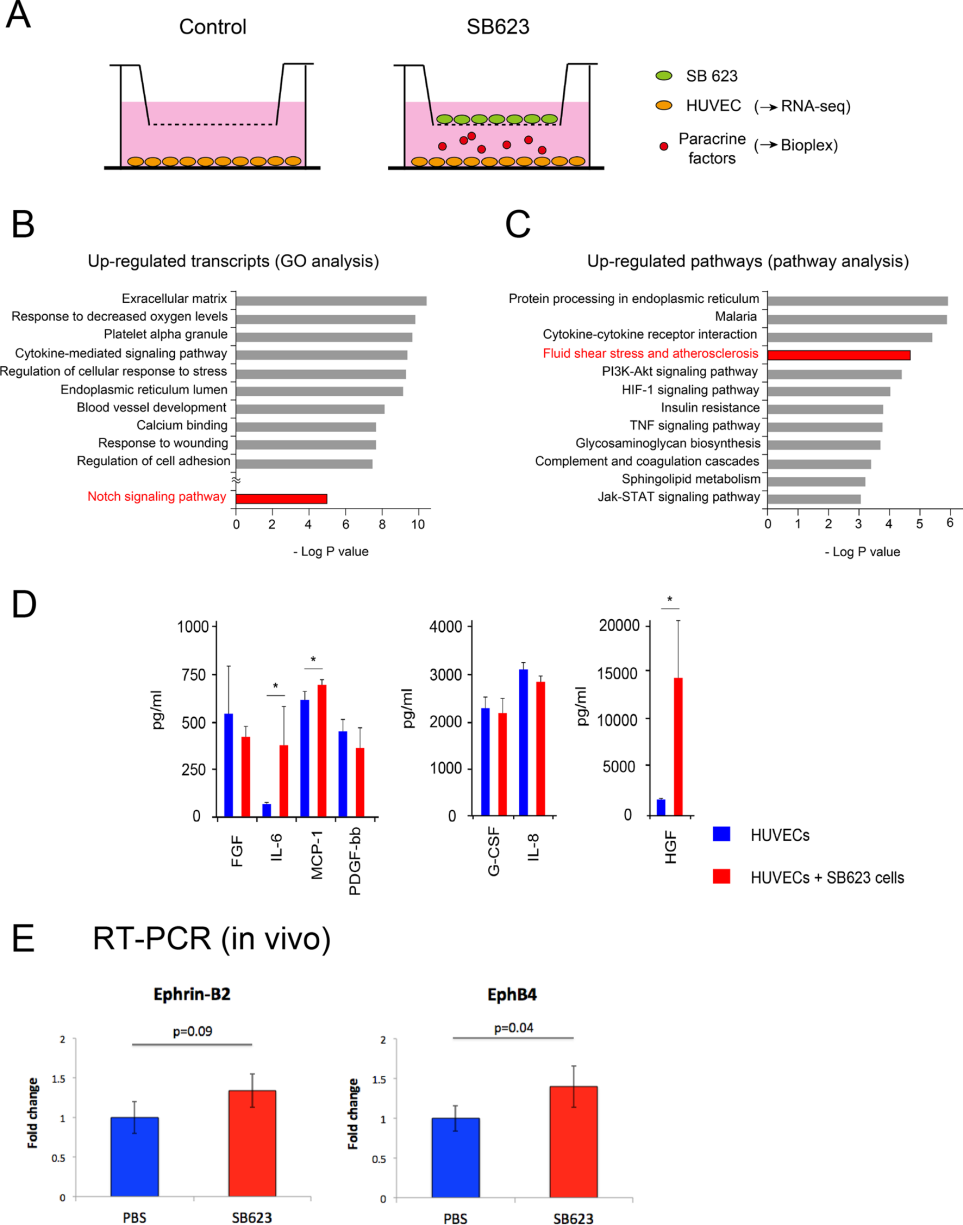
Molecular mechanism of arteriogenesis induced by SB623 cells. (**A**) Using a transwell assay, HUVECs were cocultured with SB623 cells. HUVECs were analyzed by RNA-sequencing and cell supernatants were analyzed by Bio-plex assay. (**B**) Upregulated transcripts in HUVECs cocultured with SB623 cells determined by GO term analysis. (**C**) Upregulated pathways in HUVECs cocultured with SB623 cells determined by pathway analysis. (**D**) Cell supernatants containing SB623 cells showed increased protein concentrations of IL-6, MCP-1, and HGF as compared to the control. *p < 0.05. (**E**) qPCR results from rat muscle showed gene expressions of Notch-downstream markers in the PBS and SB623 groups.

To investigate the paracrine effect of SB623 cells on endothelial cells, we performed transcriptome analysis of human umbilical vein endothelial cells (HUVECs) under hypoxia (5% O_2_) cocultured with SB623 cells using trans-well system (Fig. 4A). HUVECs were cultured without direct contact to SB623 cells and completely isolated before analysis. As a control, solely cultured HUVECs were used. A comparison of the two groups showed 3480 differently regulated genes with a false discovery rate < 0.05 (Supplementary Table S1 online). Interestingly, gene ontology (GO) analysis showed that the “Notch signaling pathway” (GO:0007219) was strongly upregulated in HUVECs co-cultured with SB623 cells, while other upregulated transcripts included “extracellular matrix” (GO:0031012), “response to decreased oxygen levels” (GO:0036293), “blood vessel development” (GO:0001568), and “response to wounding” (GO:0009611) (Fig. 4B, Supplementary Table S2 online). Previous reports have suggested that “extracellular matrix” synthesis is required during arterial maturation^15^, while “responses to decreased oxygen levels” and “responses to wounding” have been shown to be profoundly associated with angiogenesis^16,17^. In addition, pathway analysis revealed upregulation of “fluid shear stress and atherosclerosis” (hsa:05418) (Fig. 4C, Supplementary Table S3 online), suggesting that SB623 cells induced arteriogenic responses, because inflammation and shear stress are known to induce arteriogenesis via this patway^18,19^. Similarly, the “hypoxia inducible factor-1 (HIF-1) signaling pathway” was upregulated, indicating induction of angiogenic responses^16^. In addition, the “PI3K-Akt signaling pathway” (hsa:04151) and “Jak-STAT signaling pathway” (hsa:04630) were upregulated in HUVECs co-cultured with SB623 cells.

Next, cytokine concentrations of the cell supernatant in transwell system were measured (Fig. 4A). Using a Bio-plex suspension array system, cytokines associated with angiogenesis, arteriogenesis, and inflammation were screened. We found that the concentrations of hepatocyte growth factor (HGF) (*p* = 0.0004), monocyte chemoattractant protein-1 (MCP-1) (*p* = 0.004), and interleukin-6 (IL-6) (*p* = 0.004) were significantly higher in the supernatant of HUVECs cocultured with SB623 cells (Fig. 4D). In previous reports, MCP-1 was shown to be an important arteriogenic cytokine^20^ and HGF was suggested to induce arteriogenesis in a synergistic manner with other cytokines^21^. In addition, since arteriogenesis is induced by inflammation^18,22^, increased secretion of IL-6 during arteriogenic response seems to be rational.

Next, we examined arteriogenic marker gene expression in vivo. Several lines of inquiry have shown that the receptor tyrosine kinase EphB4 and its ligand ephrin-B2 play crucially important processes during arterial remodeling^8,9^. We analyzed the gene expressions of ephrin-B2 and EphB4 in muscles harvested 5 days after PBS or SB623 cell injection. While those treated with SB623 cells showed a trend for a higher level of ephrin-B2 expression as compared to the control (*p* = 0.09), EphB4 expression was significantly higher (*p* = 0.04) (Fig. 4E). In an analysis of gene expression of cytokines associated with angiogenesis, arteriogenesis, and inflammation, upregulation was not found (Supplementary Fig. S1 online).

## Discussion

In this report, we demonstrated that transplantation of Notch signaling-modified hMSCs, SB623 cells increased tissue perfusion in a rat hindlimb ischemia model. Histological findings showed an increase in the number of mature arterioles with a diameter > 15 μm. In vitro, it was revealed that paracrine factors from SB623 cells induced various pathways that potentially lead to arteriogenesis in endothelial cells. Furthermore, a trend for higher ephrin-B2 and significantly higher EphB4 expression, arteriogenic markers expressions were confirmed in ischemic limb muscles treated with SB623 cells. Therefore, we concluded that SB623 cells improved tissue perfusion by inducing arteriogenesis in the rat hindlimb ischemia model, suggesting that they would be a promising cell source for treatment of CLTI.

In this study, we showed that SB623 cell transplantation activated Notch signaling in endothelial cells in vitro, and upregulated ephrin-B2 and EphB4 in vivo. For postnatal arteriogenesis, as well as embryonic, activations of Notch signaling, ephrin-B2, and EphB4 seem to be the most important process^8–13^. Previous reports have noted that Notch activation induced ephrin-B2 expression in endothelial cells, which then mediated a sequence of vascular responses, including arterial remodeling, branching, and outward growth during the postnatal period^8,9^. Furthermore, they showed that ephrin-B2 activation induces perivascular EphB4 expression, leading to venous neovascularization. This drainage vein development likely plays an important role in improvement of tissue perfusion. In their report, it was concluded that interaction of vascular ephrin-B2 with perivascular EphB4 is essential for arterial remodeling. In the present study, we only confirmed the upregulation of these arterial and venous markers. However, others have suggested that Notch signaling also regulates more complex morphogenetic events, such as tubular sprouting, fusion, and network formation via tip cell formation as well as vascular smooth muscle cell differentiation^23,24^. Additional investigations are warranted to confirm whether SB623 cell transplantation induces arteriogenesis via Notch signaling as well as further evaluations of the beneficial effects of SB623 cells on those complex remodeling events.

In the present study, we analyzed the effects of SB623 cells on endothelial cells utilizing RNA-sequencing and several pathways considered to potentially contribute to arteriogenesis were detected. Notably, pathway analysis revealed significant upregulation of “fluid shear stress and atherosclerosis” pathway. Shear stress was a well-recognized inducer of arteriogenesis^18,19^. Increase in shear stress caused by arterial occlusion activates a cascade of events, including endothelial cell proliferation, increased permeability of endothelium, and adherence of monocytes to endothelium, consequently leading to arteriogenesis^19^. Therefore, upregulation of the fluid shear stress pathway induced by SB623 cells is suggested to be profoundly associated with arteriogenesis. Furthermore, the extracellular matrix pathway has been shown to be related to stabilization of arterial structures during arterial maturation^15^, while response to decreased oxygen level, blood vessel development, response to wounding, and the HIF-1 signaling pathway indicates an angiogenic response^16,17^. Upregulation of these pathways may play a supportive role for induction of arteriogenesis via Notch signaling because Notch signaling provides diverse functions in a context-dependent manner^25^. However, such upregulation of a broad range of pathways may be difficult to achieve by cytokine therapy or gene therapy, thus SB623 cell transplantation may be suitable for arteriogenesis induction. In the present in vivo experiment, neither angiogenic nor arteriogenic cytokine expression was upregulated, while an arteriogenic marker demonstrated upregulation. These results suggest the importance of the involvement of various pathways rather than specific cytokine upregulation, though the possible explanation by measurement timing cannot be denied. In addition, upregulation of the PI3K-Akt and Jak-STAT signaling pathways would contribute to improve cell survival^26,27^, leading to enhancement of therapeutic effects. Taken together, these results suggested that SB623 cell could be a promising cell source for arteriogenic therapy for CLTI.

This study has some limitations. First, we did not compare the efficacy of SB623 cells with that of untransfected hMSCs, thus superiority of the Notch signaling modification was not clearly shown. However, the main goal of this study was to examine the therapeutic efficacy of SB623 cells for critical limb ischemia via angiogenesis or arteriogenesis. Our positive results regarding arteriogenesis would provide a motivation for utilizing SB623 cells in CLTI patients. Second, we did not fully examine the mechanism of arteriogenesis induced by SB623 cells in in vivo study. To confirm the SB623 cell- or Notch signaling- induced arteriogenesis, further studies are needed. Immunochemistry staining of Notch signaling related protein and mature vessels at the several time points seems to be a good evaluation method.

## Conclusions

Transplantation of Notch signaling-modified hMSCs, SB623 cells improved tissue perfusion via arteriogenesis in a rat hindlimb ischemia model. The present findings suggest that SB623 cell transplantation could be one of possible therapeutic options for CLTI.

## Materials and methods

All animal protocols were approved by the Animal Experimentation Committee of Osaka University and performed according to the Guidelines for Animal Experiments of Osaka University.

### SB623 cell preparation

Notch signaling-modified hMSCs, SB623 cells were produced by transient transfection of a plasmid encoding the human Notch-1 intracellular domain cDNA to bone marrow derived hMSCs obtained from a healthy adult human donor (SanBio, Inc., Mountain View, CA, US). SB623 cells were produced under good manufacturing practice (GMP) conditions to comply with clinical use and the same cell product was examined in this study. Changes in Notch pathway profiling and responses of selected Notch target genes were ascertained, previously^28^.

### Rat hindlimb ischemia model and assessment of blood perfusion

Female Sprague–Dawley rats (10 weeks old) were purchased from SLC Japan (Shizuoka, Japan). The hindlimb ischemia model was established by removing the left femoral artery and vein^29,30^. The most distal portion of the saphenous artery was ligated, while proximally, femoral artery below the inguinal ligament was ligated. Associated side branches including the popliteal and proximal caudal femoral arteries were also ligated. Tissue perfusion was assessed with a LDPI analyzer and the LDPI index was calculated as the ratio of perfusion of ischemic hindlimb to that of healthy hindlimb. Five days after ischemic induction, rats with LPDI index > 70% were excluded, then randomly divided into the PBS injection (control) and the SB623 cells transplantation (treatment) groups. Seven days after ischemic induction, 1 × 10^5^ SB623 cells in 1 ml of PBS or 1 ml of PBS was injected into the ischemic adductor muscle at 5 different positions with a 26-gauge needle. At 7, 14, 21, and 28 days after cell transplantation, tissue perfusion was measured. For immunosuppression, 10 mg/kg/day cyclosporine was delivered using an Alzet osmotic pump (Alza Corp., Palo Alto, CA, US), which was subcutaneously implanted into each rat at 48 h before cell implantation. The implanted pumps were exchanged biweekly.

### Histological analysis

Rats were euthanized at 28 days after cell transplantation. The adductor muscle was dissected, then formalin-fixed, paraffin-embedded, and cut into 5-µm sections using a microtome for histological analysis. For assessment of capillary vessel, ischemic muscle sections were stained with sheep polyclonal anti-von Willebrand factor (vWF) antibody (AB7356, 1:50; Millipore). To assess mature arterioles, those were stained with sheep polyclonal anti-vWF and rabbit polyclonal anti-alpha smooth muscle actin (αSMA) (MO851, 1:50; Dako) antibodies, then arterioles > 15 µm were counted^21,31,32^. Furthermore, the sections were stained using hematoxylin and eosin and Sirius red. Muscle atrophy was assessed based on average fiber size, calculated as the ratio of muscle area to number of muscle fibers. The value of fiber size was expressed as the ratio of fiber of ischemic hindlimb to that of healthy hindlimb. Fibrosis was quantified using the collagen volume fraction with the Metamorph software package (Molecular Devices, Sunnyvale, California, US). Histological measurements were performed in 5 randomly selected fields of each tissue section. Obtained images were examined by optical microscopy (Keyence, Osaka, Japan).

### RNA sequencing

Using a Transwell system, HUVECs on the bottom were cocultured with SB623 cells on the top, while solely cultured HUVECs were used as the control. Following 72 h in vitro incubation under hypoxia (5% O2), HUVECs were completely isolated from SB623 cells and total RNA was extracted using an RNeasy Kit (Qiagen, Hilden, Germany), according to the manufacturer’s instructions. RNA concentration was determined with a NanoDrop ND-1000 Spectrophotometer (Thermo Fisher Scientific, Waltham, MA, USA) and RNA integrity was assessed using a Bioanalyzer 2100 (Agilent Technologies, Palo Alto, CA, USA). Next, paired-end sequencing libraries were generated from each RNA sample using an Illumina TruSeq Stranded mRNA Kit (Illumina, San Diego, CA, USA), according to the manufacturer’s protocol. Sequencing was performed on a NovaSeq6000 platform (Illumina). After quality filtering according to the FastQC, reads were trimmed using a Fastq Quality Trimmer, and mapped to the reference human genome GRCh38 using Top Hat and Bowtie 2. The abundance of Ref Seq genes was estimated using featureCounts. Differentially expressed genes between HUVECs co-cultured with BS623 cells and solely cultured HUVECs were identified using edgeR. A false discovery rate (FDR) < 0.05 was defined as significant. Gene ontology analysis was performed using Metascape (http://www.metascape.org).

### Bio-plex assay for cytokine analysis

The concentration of cytokines in cultured cell supernatants was measured with a Bio-plex system (Bio-Rad Laboratories, Hervules, CA, US), according to the manufacturer’s protocol. Two independent repetitions in duplicate were made per sample. A standard curve ranging on average from 0.15 to 3700 pg/ml (High Photomultiplier Tube Setting-PMT setting) was prepared and then fitted using the Bio-Plex Manager software package. The concentrations of basic fibroblast growth factor (FGF-2), HGF, platelet-derived growth factor-BB (PDGF-BB), stromal cell-derived factor-1α (SDF-1α), vascular endothelial growth factor (VEGF), granulocyte colony-stimulating factor (G-CSF), granulocyte–macrophage colony-stimulating factor (GM-CSF), MCP-1, interleukin (IL)-1a, IL-4, IL-6, IL-8, and IL-10 were measured.

### Real time quantitative polymerase chain reaction

Gene expressions in muscle samples were measured using a real time quantitative polymerase chain reaction (RT-PCR) assay. The adductor muscle was harvested 5 days after cell transplantation or PBS injection, then the samples were immersed in RNA (Invitrogen). Total RNA was isolated using an RNeasy Kit (Qiagen, Hilden, Germany) and then reverse-transcribed using an Omniscript Reverse Transcriptase kit (Qiagen). RT-PCR was performed using TaqMan Gene Expression Assay Master Mix (Applied Biosystems, California, US) with the 7500 Fast Real-Time PCR System (Applied Biosystems). Primers used in this study were as follows: ephrin-B2 (Assay ID: Rn01756899_m1), EphB4 (Assay ID: Rn01481051_m1), and GAPDH (Assay ID: Rn01775763_g1).

### Statistical analysis

All continuous variables are summarized as mean ± standard deviation and were compared using Student’s *t*-test. All *p *values are two-sided, with those < 0.05 considered statistically significant. All statistical analyses were performed using JMP Pro14 (SAS Institute, Cary, NC).

## Supplementary Information

Supplementary Information.

Supplementary Table S1.

Supplementary Table S2.

Supplementary Table S3.

## Data Availability

The datasets of this study are available from the corresponding author upon reasonable request.
