# Supplementary material for: Notch signaling-modified mesenchymal stem cells improve tissue perfusion by induction of arteriogenesis in a rat hindlimb ischemia model
Source: Sci Rep. 2021 Jan 28;11:2543. doi: 10.1038/s41598-021-82284-3 (PMC7844258; doi:10.1038/s41598-021-82284-3)

**Supplemental information**

**Notch Signaling-modified Mesenchymal Stem Cells**

**Improve Tissue Perfusion by Induction of Arteriogenesis**

**in a Rat Hind Limb Ischemia Model**

Shusaku Maeda^1^, Shigeru Miyagawa^1^, Takuji Kawamura^1^, Takashi Shibuya^1^, Kenichi Watanabe^1^, Takaya Nakagawa^1^, Akima Harada^1^, Dai Chida^2^, Yoshiki Sawa^1 *^

Institutions and affiliations:

^1^Department of Cardiovascular Surgery, Osaka University Graduate School of Medicine, Suita, Osaka, Japan

^2^SanBio, Inc., Tokyo, Japan

**Figure S1. qPCR results of rat muscle showing cytokine gene expressions**

**Table S1. Differences in gene expression between HUVECs cocultured with SB623 cells and solely cultured HUVECs**

H1-3: solely cultured HUVECs (control), S1-3: HUVECs cocultured with SB623 cells

FDR, false discovery rate; Log FC, log fold-change

**Table S2. Upregulated transcripts in HUVEC cocultured with SB623 cells analyzed by GO analysis**

GO: gene ontology

**Table S3. Upregulated transcripts in HUVEC cocultured with SB623 cells analyzed by pathway analysis**

KEGG: Kyoto Encyclopedia of Genes and Genomes

**Figure S1.** qPCR results of rat muscle showing cytokine gene expressions. Cytokines associated with angiogenesis, arteriogenesis, and inflammation were examined, with no significant upregulation.


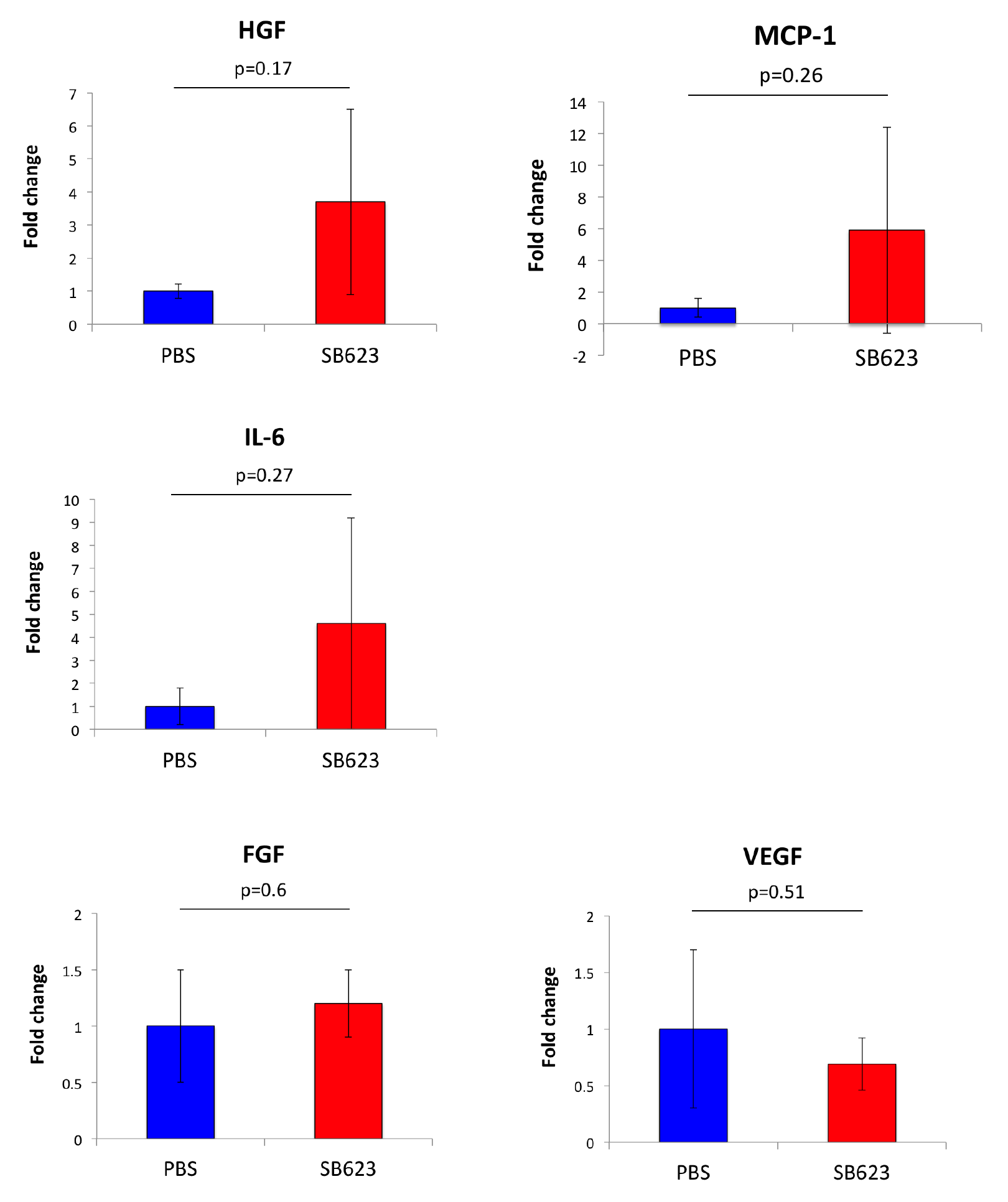

Supplement: Supplementary file 1 — Supplementary Information. [file 41598_2021_82284_MOESM1_ESM.docx]
